# Rise and Growth of Vaccination Law.—V

**Published:** 1904-02-13

**Authors:** J. E. R. Stephens

**Affiliations:** author of "Digest of Public Health Cases," etc., etc.


### HOSPITAL ADMINISTRATION.

### CONSTRUCTION AND ECONOMICS.

### RISE AND GROWTH OF VACCINATION LAW.—V.

By J. E. R. STEPHENS, Barrister-at-law, author of "Digest of Public Health Cases," etc., etc.

(*Continued from page* 324).

MEANS FOR DIMINISHING SMALL-POX.

THE Royal Commissioners on Vaccination in their report further stated that in order to maintain in efficiency the primary essential condition of a system of isolation, viz, the immediate isolation of a person attacked by the disease, it is requisite to have a hospital always ready, with sufficient accommodation for the reception of all such cases, and there are no means of estimating what extent of accommodation will suffice to meet at all times the necessities of a particular town. It is certain that the disease spreads more rapidly, its contagion seems to operate more actively, at one time than at another. If an epidemic affects a locality, the preparations made for the isolation of small-pox cases, which have proved to be fully adequate in ordinary years, may turn out to be quite inadequate. It is impossible at once to provide the needed hospital accommodation. If the cases are to be removed to a hospital at all, the massing of large numbers together, in itself a means of spreading the disease, might prove inevitable. "We have only to look at what happened at Leicester to see how suddenly the necessities of the case may outrun the preparations made for isolation. Moreover, although the vaccination of children had been neglected in Leicester for many years, it would be quite a mistake to regard it as an unvaccinated town. The population over 20 years of age was probably well vaccinated, and a large proportion of those between 10 and 20 years of age were vaccinated persons. More than half even of those between seven and ten years of age at the commencement of the epidemic must be placed in the same category."

"We can see nothing to warrant the conclusion that in this country vaccination might safely be abandoned and replaced by a system of isolation. If such a change were made in our method of dealing with small-pox, and that which had been substituted for vaccination proved ineffectual to prevent the spread of the disease (it is not suggested that it could diminish its severity in those attacked), it is impossible to contemplate the consequences without dismay. Power should, in our opinion, be conferred on sanitary authorities to give compensation for loss of wages, and generally pay expenses occasioned either by the isolation of patients, or persons who have come in contact with them, or such supervision of them as is necessary, whether in hospital or elsewhere."

The attention of the Commissioners was drawn to the circumstance that outbreaks of small-pox have not unfrequently had their origin in the introduction of the disease to common lodging-houses by tramps wandering from place to place. In view of this they made the following recommendations:—

i. That common shelters which are not now subject to the law relating to common lodging-houses should be made subject to such law.

ii. That there should be power to the local authority to require medical examination of all persons entering common lodging-houses and casual wards to see if they are suffering from small-pox, and to offer a reward for prompt information of the presence of the disease.

iii. That the local authority should have power to order the keeper of a common lodging-house in which there has been small-pox to refuse fresh admissions for such time as may be required by the authority.

iv. That the local authority should be empowered to require the temporary closing of any common lodging-house in which small-pox has occurred.

v. That the local authority should have power to offer free lodgings to any inmate of a common lodging-house or casual ward who may reasonably be suspected of being liable to convey small-pox.

vi. That the sanitary authority should give notice to all adjoining sanitary authorities of the occurrence of small-pox in common lodging-houses or casual wards.

vii. That where the disease occurs, the public vaccinator or the medical officer of health should attend and vaccinate the inmates of such lodging-houses or wards, except such as should be unwilling to submit themselves to the operation.

"In connection with the subject with which we have been dealing," say the Commissioners, "we may advert to the suggestion that the vaccination and the sanitary authority should in all cases be identical. . . . Indeed, the advantage of placing in the same hands the supervision of vaccination and of the other proposal to do so deserves most serious consideration. Under present arrangements, however, such a proposal raises very great difficulties. Whilst in England and Wales there are only 648 vaccination districts, the sanitary authorities exceed 1,700 in number. . . . Many instances might be cited to show that it would be impracticable to vest the sanitary and vaccination duties in all cases in a single local authority without a complete recasting of our present areas of local administration. We are not in a position to devise a scheme which would accomplish, either wholly or partially, the desired result. At the same time we fully recognise the importance of achieving it as far as possible, and we should regard with favour such changes as would render the amalgamation of the vaccination and sanitary authorities feasible, or indeed any steps taken in that direction, even although they should only partially effect the object in view."

The third question put to the Commissioners was as to the objections made to vaccination on the ground of injurious effects alleged to result therefrom, and the nature and extent of any injurious effects which do, in fact, so result.

In the first instance the majority of the Commissioners considered the evidence afforded by infant mortality. Vaccination is, in the vast majority of cases, coincident in point of time with infancy. "If, then," say the Commissioners, "it is the parent of other diseases, and has substantially augmented the number of deaths due to them, we should expect to see some effect produced on infant mortality as a whole; yet it is clear that the mortality of infants in the first year of life, as measured by the proportion of their deaths to births, has not increased at all during the times when infant vaccination has been increasing."

"If vaccination were, to any serious extent, a cause of syphilis we should have expected to see some evidence of it in the comparative records of the mortality of infants under one year of age. Yet we find that whereas in England and Wales there was, as between the periods 1863–1867 and 1883–1887, an increase in the infant mortality from syphilis in England and Wales of only 24·7 per cent., the increase between the same periods in Leicester was no less than 69·3 per cent. This of course does not imply any connection between the disuse of vaccination and the increase of infantile syphilis. It does, however, conclusively rebut the argument of those who seek to connect the increase of mortality from syphilis with the practice of vaccination. . . . Even if it can be shown that in some instances syphilis has been inoculated by vaccination, the conclusion would still remain that this cannot have been so to any substantial extent."

In regard to cancer the report says:—

"It may be that, in addition to the apparent increase, there has been some real increase in the mortality from cancer; but there is not a shadow of evidence to connect this with the practice of vaccination, whilst there is . . . evidence pointing the other way."

As regards erysipelas the Committee say that "the evidence is, in our opinion, conclusive to show that there has not been during the last forty years any material increase of deaths from erysipelas owing to vaccination."

### Personal Injury or Death from Vaccination.

The allegation that personal injury or death has resulted from vaccination is next dealt with (paragraphs 406–434).

"From all sources 421 cases in which death or non-fatal injury has been alleged or suggested to have been connected with vaccination have been brought to our notice from June 1st, 1889, to July 1st, 1896. These 421 cases, however, include 19 groups of connected cases, each of which has only been counted as one in arriving at that number. The individual cases included in these groups amount to about 150. Some of these 421 cases were investigated and made the subject of reports by medical inspectors of the Local Government Board.

"We (the Committee) received reports with reference to a large number of them from medical men appointed by ourselves. In a few cases the nature of the allegation or suggestion rendered it unnecessary, in our opinion, to make any inquiry into the case. In a considerable number we sought for further information, and after we had considered the further facts thus acquired there appeared to be no necessity for an investigation by the medical men who assisted us by personally inquiring into cases of alleged injury from vaccination.

"We have not any means of ascertaining in what number of cases some other disease has supervened on vaccination, as a consequence of it, without producing a fatal result. We are able, however, to form some judgment upon this point by observing the number of non-fatal cases to which our attention has been called. A consideration of all the circumstances has led us to the conclusion that, as regards the non-fatal cases with which we are now dealing, serious injury cannot have resulted in any considerable number of cases."

An examination of the analysis of the fatal maladies connected with vaccination during the period 1886 to 1891, made by Dr. Ogle, shows that erysipelas is credited with almost one-half of the total number of deaths. To these a considerable number is to be added where inflamed arms occurred, but in which the disease did not receive the name of erysipelas, though it was probably allied to it. Next in number comes the class which includes pyæmia, septicæmia, and blood-poisoning. If this class be added to erysipelas and maladies allied to it, they account altogether for two-thirds of the cases in which the cause of death has been connected with vaccination. An examination of the particulars of the cases of alleged deaths and injury from vaccination, to which the attention of the Commissioners was called during the last six years, shows that the death or injury has been attributed in the great majority of cases to one or other of these diseases, and chiefly to erysipelas.

It must not be forgotten that the introduction into the system of even a mild virus, however carefully performed, is necessarily attended by the production of local inflammation and of febrile illness. If these results did not in some measure follow, the practice would probably fail in its protective influence. As a rule, the inflammation and illness are of a trifling character; in exceptional cases, however, they may exhibit more severity, and, as certain facts submitted to us, the Commissioners, in evidence have shown, there are cases—though these are rare—where a general eruption may follow vaccination.

It is not always easy to determine whether vaccination has been the cause of, or has contributed to, subsequent erysipelas or blood-poisoning. Erysipelas is a common disease in infancy, and not unfrequently leads to death. The evidence of Dr. Ogle shows that nearly two thousand per million die of erysipelas during the first three months of life, and that the mortality rapidly declines as the age advances. Quite apart, then, from vaccination, there is nothing remarkable in the occurrence of erysipelas in the case of an infant. The disease is obviously contracted, in the majority of cases, from some other source. Where a child has been in good health prior to vaccination, and is seized with any malady after it, it is not unnatural that the two occurrences should be connected together as cause and effect by those who have not a wide experience of the liability to be attacked by the disease independently of vaccination. There can be no doubt that even very slight wounds may lead to erysipelas. It has been induced by scratches from pins, abrasions from the dress, and other injuries in themselves most trivial.

A careful examination of the facts which have been brought under the notice of the Commissioners enabled them to arrive at the conclusion that, although some of the dangers said to attend vaccination are undoubtedly real, and not inconsiderable in gross amount, yet when considered in relation to the extent of vaccination done they are insignificant. There is reason further to believe that they are diminishing under the better precautions of the present day, and with the addition of the further precautions which experience suggests will do so still more in the future.

### Vaccination with Animal Vaccine.

The fourth question investigated by the Commissioners was whether any, and if so what, means should be adopted for preventing or lessening the ill effects, if any, resulting from vaccination; and whether, and if so by what means, vaccination with animal vaccine should be further facilitated as a part of public vaccination.

In reply to this question the majority recommend: the use of calf lymph, because "this would afford an absolute security against the communication of syphilis;" that the age limit for vaccination should be extended to six months; that any abrasion of the vaccination vesicles by clothing of a nature likely to irritate them should be avoided, and foreign substances should not be rubbed into the wounds under circumstances calculated to set up inflammation (it is most important, too, that any rags or other materials applied to the place of vaccination should be scrupulously clean); that children should be vaccinated and inspected, as a rule, at their own homes; that lymph used should be preserved in tubes instead of on dry points (each tube should contain only sufficient lymph for the vaccination of one person); that no instrument should be used for the operation which has not been boiled or otherwise sterilised for the purpose; that the insertion of lymph in the child's arm should not be placed too close together.

After the report of the Commission had been published there was an increase of calf-lymph vaccination. On March 15th, 1898, Mr. Chaplin, President of the Local Government Board, introduced a Bill into the House of Commons for amending the law relating to vaccination. On August 12th of the same year the Bill received the Royal Assent. The new Act embodies many, though by no means all, the recommendations of the Royal Commission. By it vaccination with glycerinated calf lymph is substituted for arm-to-arm vaccination. A parent who conscientiously believes that vaccination would be prejudicial to the health of his child may escape penalty for not procuring the child's vaccination by satisfying two justices or a stipendiary or metropolitan police magistrate in petty sessions of his conscientious belief, and delivering a certificate to that effect to the vaccination officer.

